# Evaluation and Dissemination Strategies for Community-Engaged Research

**DOI:** 10.1093/geroni/igaf122.1274

**Published:** 2025-12-31

**Authors:** Sarah Dys, Liza Behrens, Elinor Schoenfield

## Abstract

Community-engaged research and evaluation strategies have not been widely discussed or documented, especially those focused on older adults and the communities in which they live. To address the needs of the gerontological research community, this symposium describes evaluation, measurement, and dissemination strategies commonly used in community-engaged research and evaluation through real world examples. Three presentations highlight diverse strategies to perform community-engaged research, conduct evaluations with community partner input, employ meaningful measurement practices, and share findings back with community partners involved in the work. The first presentation showcases multi-level evaluation of a unique community-based and intergeneration nutrition program in New York City. This program emphasizes the intersection of multisector partnerships, multiple methodologies, and a combination of participatory engagement, process evaluation, and outcomes assessments. The second presentation showcases a Patient Centered Outcomes Research Institute (PCORI) project to develop a comprehensive tool to measure engagement activities in community-engaged research and evaluation and how these activities impact research outcomes. The third presentation focuses on dissemination activities in community-engaged work, primarily working with a community-based advisory board to develop a communication and dissemination plan at the outset of a dementia care needs and assets assessment among African immigrant community members in Minnesota. Finally, the session concludes with a moderated discussion to provide a forum for participants and presenters to co-construct practice-based strategies to apply to their own community-engaged research and evaluation projects centering older adults and the communities in which they live. Participants are encouraged to engage in discussion to generate applications for future dissemination. Community-Engaged Research Interest Group Sponsored Symposium

